# Frequency of Acute Viral Hepatitis A, B, C, and E in Pregnant Women Presenting to Hayatabad Medical Complex, Peshawar, Pakistan

**DOI:** 10.7759/cureus.24208

**Published:** 2022-04-17

**Authors:** Ahmad R. Khan, Salma Waqar, Zainab Rafiq, Rizwan Ullah, Muhammad Hayyan Wazir, Ayesha M Gul

**Affiliations:** 1 Internal Medicine, Hayatabad Medical Complex Peshawar, Peshawar, PAK; 2 Internal Medicine, Rehman Medical Institute, Peshawar, PAK; 3 Gynaecology, Mardan Medical Complex, Mardan, PAK

**Keywords:** hepatitis infection in pregnancy, hepatitis e (he-igm), anti-hepatitis c virus (anti-hcv), hepatitis b surface antigen (hbsag), hepatitis a, frequency-acute viral hepatitis

## Abstract

Objective

To determine the frequency of hepatitis A, B, C, and E viruses (HAV, HBV, HCV, HEV) in pregnant women with acute hepatitis presenting to the medical units of HMC, Peshawar.

Methods

This is a descriptive cross-sectional study in which 442 pregnant women, either multi or primigravida, with yellow discolouration of the sclerae of the eyes and elevated alanine aminotransferase (ALT) > 100 U/L on laboratory tests were chosen by convenience (non-probability) sampling to assess the frequency of HAV, HBV, HCV, HEV.

Results

The majority, i.e., 58.8%, were in the age range of >30 years and presented in the third trimester. Of the subjects, 81.90% had parity in the range of 1-5, 89.4% were multigravida, 71.3% were illiterate, and 73.1% were in the low-income category. Anti-HEV was detected in 47.3% of pregnant women, Anti-HCV in 30.3%, Hepatitis B surface antigen (HBsAg) in 11.5%, Hepatitis A-IgM in 5%, and 5.90% of the cases were virus-free.

Conclusions

HBV, HCV, and HEV exposure, in particular, may have a substantially larger impact on pregnancy and neonatal outcomes than HAV. As a result, at the first prenatal appointment, standard viral hepatitis screening in pregnant women may need to be reviewed.

## Introduction

The most prevalent cause of jaundice in pregnancy is acute viral hepatitis. Most viral hepatitis infections (e.g., hepatitis A, B, C, and D) are unaffected by pregnancy; nevertheless, patients with hepatitis E virus (HEV) infection have seen a more severe course of viral hepatitis during pregnancy. Opinions, however, differ on the maternal and fetal outcomes of pregnancy-associated viral hepatitis. The majority of women with hepatitis will have a normal pregnancy; however, the physical process of pregnancy may cause certain liver disorders. Gallstones (or "cholelithiasis") can develop in about 6% of women with hepatitis during pregnancy [1-3].

A study from Bangladesh reported that acute HEV infection in the third trimester of pregnancy and HEV-induced fulminant hepatic failure were associated with 80% of mortality despite the best possible care. Acute hepatitis E is the major cause of a wide range of liver diseases in Bangladesh, ranging from severe acute viral hepatitis to fulminant hepatic failure and decompensation of liver cirrhosis [4]. Pregnant women with jaundice and acute viral hepatitis caused by HEV infection had a higher maternal mortality rate and worse obstetric and fetal outcomes than pregnant women with jaundice and acute viral hepatitis caused by other types of hepatitis viruses [5].

It has been documented that viral hepatitis during pregnancy is associated with a significant risk of maternal problems, has a high incidence of vertical transmission resulting in foetal and neonatal hepatitis, and is a major cause of maternal death [4]. The global prevalence of hepatitis C virus (HCV) infection in pregnant women is predicted to be 1-8%, while in children it is predicted to be 0.05-5%. While parenteral transmission is still significant in developing countries, perinatal transmission is currently the most common method of transmission of HCV in developed countries. Vertical transmission cannot be prevented because there is no HCV vaccination or approved drug for use during pregnancy. Due to a low vertical transmission rate of 3-5%, a high rate of spontaneous clearance (25-50%), and delayed morbidity, HCV has been overlooked in pregnant women and their babies [5].

In a study done in the Obstetrics and Gynecology Department of Peoples Medical College in Nawabshah, Sindh, Pakistan, HCV antibodies were discovered in 102 (3.44%) of the 3020 pregnant women studied. Using polymerase chain reaction (PCR), 73 (71.52%) of them tested positive for HCV-RNA. Of the anti-HCV positive women, eight (7.84%) also tested positive for Hepatitis B e antigen (HBeAg) [6]. Perinatal and early childhood transmission may account for more than a third of chronic infections in low-endemic areas. Hence, it is critical to prevent Hepatitis B virus (HBV) transmission during pregnancy by identifying Hepatitis B surface antigen (HBsAg)-positive mothers and administering immunoprophylaxis to their babies [7]. The CDC advised prenatal screening for HBsAg in all pregnant women after selective screening of high-risk pregnant women for HBsAg failed to identify a substantial proportion of HBV-infected mothers. Prenatal screening of pregnant women for HBsAg is now nearly universal in the United States. However, routine screening of pregnant women for HBsAg is yet to be implemented in some other countries' prenatal care programmes, and even in Western countries, some pregnant women have never been tested for HbsAg [8].

Despite the fact that HCV affects a large number of pregnant women, little study has been done on the effects of HCV on pregnancy outcomes. Prior research on HCV and pregnancy has focused on vertical virus transmission rather than the effects of chronic HCV infection on the mother's health, delivery complications, and newborn health. Because the discovery of negative outcomes might have an impact on existing screening recommendations, this knowledge could have far-reaching public health implications. Also, pregnant women who had jaundice and acute viral hepatitis due to HEV infection had a greater maternal mortality rate and worse obstetric and fatal outcomes than pregnant women who had jaundice and acute viral hepatitis due to other kinds of hepatitis [9].

## Materials and methods

We conducted a cross-sectional study at the Department of Medicine, Hayatabad Medical Complex, Peshawar, a tertiary care hospital located in the suburb of Peshawar in Khyber Pakhtunkhwa, Pakistan. The type of study was descriptive cross-sectional and was performed over six months from September 2021 to March 2022, during which certain procedures such as data collection, analysis and results interpretation were carried out. A total of 442 pregnant women having acute hepatitis were selected for the study. The sampling technique used was convenience (non-probability) sampling and the sample size was calculated using a 95% confidence coefficient, 3.44% prevalence of hepatitis C in pregnant women [6], and 1.7% margin of error, and was calculated with the G*Power sample size calculator. Prior to our study, a No Objection Certificate (NOC) was issued for the research group to allow them to conduct their research in the above-mentioned hospital. The team of researchers in charge of data collection explained the objectives of the research to the participating patients and emphasized that participation was voluntary. Informed consent was obtained from each respondent and confidentiality was assured. Ethical clearance was taken for the study from Khyber Girls Medical College, Peshawar, Pakistan (3738 dated September 1, 2020).

Subjects were selected based on pregnancy, and clinical and biochemical evidence of hepatitis. All those pregnant women, either multi or primigravida with yellow discolouration of the sclera of the eyes and raised alanine aminotransferase (ALT) > 100 IU/L on laboratory tests were included in the study group. Excluded from the study group were those participants found to have acute presentations of non-viral chronic liver disease as this changed the pattern of our study. Patients with other concomitant liver diseases such as liver abscess, hydatid, or other cysts were excluded from our study to minimize bias. There were other notable confounders namely pregnant women with hemolytic anaemia and acute fatty liver of pregnancy who presented with jaundice and thus were excluded from the study group.

Viral hepatitis was diagnosed by enzyme-linked immunoassay (ELISA) using anti-Hepatitis A virus (HAV), HBsAg, Anti-HCV, and Anti-HEV in the study group patients. The frequency of each was then tabulated.

## Results

**Figure 1:**
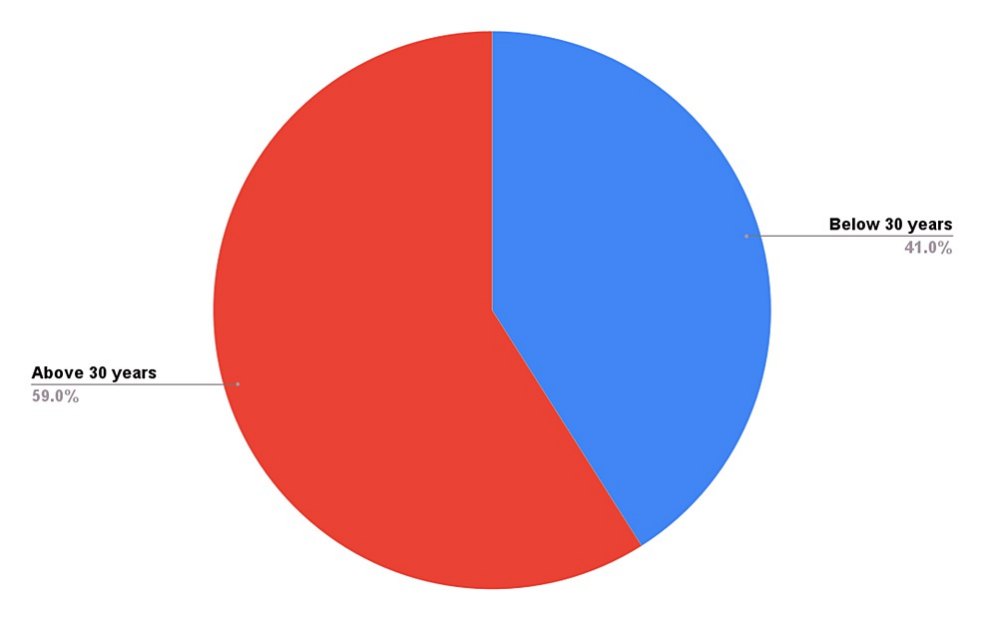
Age-wise distribution of patients

This study was performed on 442 pregnant women with clinically suspected acute hepatitis (and was confirmed by ALT/serum glutamic pyruvic transaminase (SGPT) and bilirubin levels) presenting to the out-patient department (OPD) and accident and emergency department of this institute. They were admitted to medical wards for further investigations as per inclusion criteria. The minimum age in this study was 17 years and the maximum was 50 years, with a mean age of 32.0249 + 8.3773 years (Figure 1).

The gestational age of the women at presentation ranged from the first trimester (from 1-12 weeks) to the third trimester (25-38 weeks). A total of 212 (47.97%) patients presented in the third trimester (25-38 weeks), followed by 150 (33.93%) patients presenting in the second trimester (13-24 weeks), and 80 (18.10%) patients in the first trimester (1-12 weeks). The distribution of patients showed that the majority, i.e., 362 (81.90%), had parity in the range of 1-5 and 80 (18.10%) had parity in the range of 6-10. The minimum parity in this study was 1 and the maximum was 9, with a mean parity of 3.39 + 1.99 (Table 1).

**Table 1:** Parity-wise distribution of patients (n = 442) Minimum parity = 01; Maximum parity = 09; Mean parity = 3.39 ± 1.99

Parity	Frequency	Percentage (%)
01-05	362	81.9
06-10	80	18.1
Total	442	100

**Figure 2:**
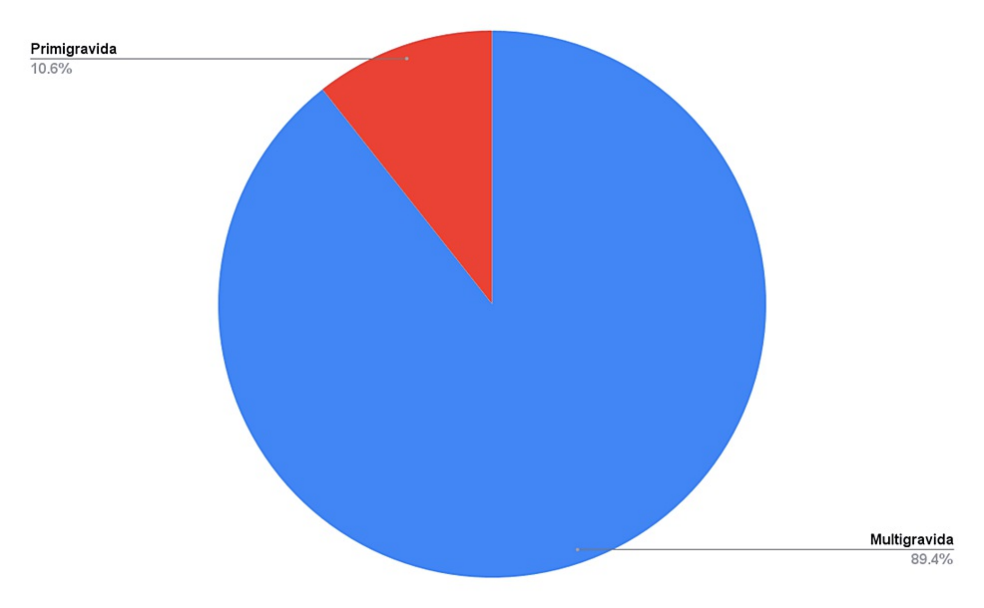
Gravidity of patients

The majority of patients were multigravida, while the remaining were primigravida (Figure 2).

The educational status of patients was tabulated, and ranged from Illiterate to Higher Secondary level (Table 2).

**Table 2:** Educational status of patients (n = 442)

Educational level	Frequency	Percentage (%)
Illiterate	315	71.3
Matric	60	13.6
Middle	32	7.2
Primary	28	6.3
Higher Secondary	7	1.6
TOTAL	442	100

The socioeconomic status of pregnant women was determined by their monthly household income. Patients with an income of less than Pakistani rupee (PKR) 25,000 per month were included in the Poor category, whereas those with an income of PKR 25,000 to PKR 100,000 per month were included in the Middle category (Table 3).

**Table 3:** Socio-economic status of patients ( n = 442)

Socioeconomic status	Frequency	Percentage (%)
Poor	323	73.1
Middle	119	26.9
Total	442	100

Laboratory investigations revealed that in the majority of 378 (85.5%) pregnant women, alanine aminotransferase (ALT) was elevated and serum bilirubin was also raised in the majority of 380 (86%) patients (Table 4).

**Table 4:** Laboratory report of alanine aminotransferase (ALT) and serum bilirubin in patients (n=442)

Laboratory Report	Frequency	Percentage (%)
Elevated ALT level	378	85.5
Elevated Serum Bilirubin Levels	380	86

Hepatitis E (Anti HEV) was discovered in 209 (47.3%) pregnant women based on viral profiles. HBsAg were detected in 51 (11.5%) of the patients, whereas Hepatitis A-IgM was detected in 22 (5%) of the cases. There were 26 (5.90%) instances that were virus-free (Table 5).

**Table 5:** Frequencies of viruses in patients (n=442)

Viruses	Frequency	Percentage (%)
Hepatitis E virus	209	47.30
Hepatitis C virus	134	30.30
Hepatitis B virus	51	11.50
Hepatitis A virus	22	5.00
Virus free (Toxic hepatitis, Autoimmune)	26	5.90
Total	442	100

## Discussion

Hepatitis caused by a virus during pregnancy is linked to a higher risk of complications for the mother. According to reports, it has a significant risk of vertical transmission and is the primary cause of maternal mortality [9]. Hepatitis A, B, C, D, and E viruses can all cause viral hepatitis. Hepatitis A, B, and C do not appear to affect the course of pregnancy in the Western world, but HEV infection, when obtained during the second or third trimester, appears to have a greater chance of developing into fulminant hepatitis [10]. Transmission of Hepatitis A from mother to child appears to be quite rare.

HEV causes high mortality in women infected by the virus during pregnancy (10-25%) [11,12]. HEV infection in pregnant women is more common and fatal in the third trimester. The prevalence of HEV infection in the second and third trimesters of pregnancy (19.4% and 18.4%, respectively) was found to be much higher than in the first trimester (8.8%). The fatality rate in pregnant women with fulminant hepatic failure has also been reported to be very high, i.e. 22.2% with maximum severity reported in the third trimester (44.4%) [13,14]. Prevalence of HEV IgG was found to be 0.6-2% and 12.6% in pregnant women in Spain and Turkey, respectively [15-17]. In an Indian study, out of the 300 asymptomatic pregnant women studied, 101 (33.67%) tested positive for anti-HEV gG antibodies [18]. When Anti-HEV seroprevalence was compared to age, an increasing trend was observed but the result was not significant.

Our study's findings also showed a higher prevalence of anti-HEV IgG (47.3%) in pregnant women than in other similar studies in which a frequency of (2-13%) was reported [15-17]. An Indian study showed that pregnant women are susceptible to HEV infection in early pregnancy and the probability of exposure to HEV during pregnancy was higher in urban (slum areas) than in rural populations [18]. The prevalence of anti-HEV IgG was significantly higher in the urban population because people use drinking water sources other than tap water, such as wells, and ponds. Interestingly, increasing age was not associated with the increased prevalence of anti-HEV IgG. Socio-economic status appeared to be the risk factor for anti-HEV IgG in pregnant women. Health maintenance strategies such as improved personal and public hygiene education have been shown to be successful in preventing the spread of HEV infection. If implemented early, these preventive actions may reduce maternal and neonatal mortality and morbidity from HEV infection.

## Conclusions

On the basis of the results of the study, it is concluded that acute hepatitis A, B, C, and E were found in 5%, 11.5%, 30.3%, and 47.3% of cases, respectively. The majority of pregnant women (58.8%) were in the age range of 30 years and older. The majority of pregnant women were found in the third trimester and were seen to be multigravida, illiterate, with a parity of 1-5, belonging to rural areas, and the lower socioeconomic class of society. Acute viral hepatitis, especially that caused by HBV, HCV, and HEV exposure, may have a much greater effect on pregnancy and neonatal outcomes than HAV. Routine viral hepatitis screening in pregnant women may need to be reconsidered at the first antenatal visit.
